# The Role of Antibiotic-Target-Modifying and Antibiotic-Modifying Enzymes in *Mycobacterium abscessus* Drug Resistance

**DOI:** 10.3389/fmicb.2018.02179

**Published:** 2018-09-12

**Authors:** Sakshi Luthra, Anna Rominski, Peter Sander

**Affiliations:** ^1^Institute of Medical Microbiology, University of Zurich, Zurich, Switzerland; ^2^National Center for Mycobacteria, Zurich, Switzerland

**Keywords:** non-tuberculous mycobacteria, *Mycobacterium abscessus*, antibiotic, drug resistance, resistance genes, antibiotic-target-modifying enzymes, antibiotic-modifying enzymes

## Abstract

The incidence and prevalence of non-tuberculous mycobacterial (NTM) infections have been increasing worldwide and lately led to an emerging public health problem. Among rapidly growing NTM, *Mycobacterium abscessus* is the most pathogenic and drug resistant opportunistic germ, responsible for disease manifestations ranging from “curable” skin infections to only “manageable” pulmonary disease. Challenges in *M. abscessus* treatment stem from the bacteria’s high-level innate resistance and comprise long, costly and non-standardized administration of antimicrobial agents, poor treatment outcomes often related to adverse effects and drug toxicities, and high relapse rates. Drug resistance in *M. abscessus* is conferred by an assortment of mechanisms. Clinically acquired drug resistance is normally conferred by mutations in the target genes. Intrinsic resistance is attributed to low permeability of *M. abscessus* cell envelope as well as to (multi)drug export systems. However, expression of numerous enzymes by *M. abscessus*, which can modify either the drug-target or the drug itself, is the key factor for the pathogen’s phenomenal resistance to most classes of antibiotics used for treatment of other moderate to severe infectious diseases, like macrolides, aminoglycosides, rifamycins, β-lactams and tetracyclines. In 2009, when *M. abscessus* genome sequence became available, several research groups worldwide started studying *M. abscessus* antibiotic resistance mechanisms. At first, lack of tools for *M. abscessus* genetic manipulation severely delayed research endeavors. Nevertheless, the last 5 years, significant progress has been made towards the development of conditional expression and homologous recombination systems for *M. abscessus*. As a result of recent research efforts, an erythromycin ribosome methyltransferase, two aminoglycoside acetyltransferases, an aminoglycoside phosphotransferase, a rifamycin ADP-ribosyltransferase, a β-lactamase and a monooxygenase were identified to frame the complex and multifaceted intrinsic resistome of *M. abscessus*, which clearly contributes to complications in treatment of this highly resistant pathogen. Better knowledge of the underlying mechanisms of drug resistance in *M. abscessus* could improve selection of more effective chemotherapeutic regimen and promote development of novel antimicrobials which can overwhelm the existing resistance mechanisms. This article reviews the currently elucidated molecular mechanisms of antibiotic resistance in *M. abscessus*, with a focus on its drug-target-modifying and drug-modifying enzymes.

## Introduction

Non-tuberculous mycobacteria (NTM) encompass all species of mycobacteria that do not cause tuberculosis (TB) or leprosy (Lee et al., 2015). NTM are ubiquitous in the environment and occasionally infect humans with various predisposing conditions like cystic fibrosis, bronchiectasis or immunosuppression, causing a variety of pathological conditions including pulmonary, skin and soft tissue infections and disseminated diseases (Chan and Iseman, 2013). The last few decades saw an alarming increase in the incidence and prevalence of NTM-pulmonary disease throughout the globe. Importantly, more than 90% of all reported NTM-pulmonary disease cases involved infections with the *Mycobacterium avium* complex (comprising of *M. avium*, *Mycobacterium chimaera* and *Mycobacterium intracellulare*) or *Mycobacterium abscessus*, accentuating the medical importance of these emerging pathogens (Hoefsloot et al., 2013; Ryu et al., 2016; Wu et al., 2018). For a long while, it was believed that infections with genetically diverse NTM strains were exclusively acquired upon exposure to the environment. However, a recent whole genome analysis of more than 1000 *M. abscessus* clinical isolates from different geographical locations, revealed the presence of genetically clustered strains in patients. This suggests a human-to-human transmission of *M. abscessus*, presumably through indirect mechanisms like cough aerosols or surface contamination (Bryant et al., 2016).

*Mycobacterium abscessus* is an opportunistic pathogen, ubiquitous in the environment, that often causes infections in humans with compromised natural defenses such as patients with cystic fibrosis or other chronic lung diseases (Sanguinetti et al., 2001; Brown-Elliott and Wallace, 2002; Howard, 2006; Griffith et al., 2007). A current taxonomic classification suggests separation of *M. abscessus* into three distinct subspecies: *M. abscessus* subsp. *abscessus*, *M. abscessus* subsp. *bolletii*, and *M. abscessus* subsp. *massiliense* (Tortoli et al., 2016). Although a saprophyte in water and soil, following lung infection *M. abscessus* can swiftly grow and survive intra-cellularly within macrophages as well as in extra-cellular caseous lesions and airway mucus (Wu et al., 2018). Several factors contribute to the success of this rapidly growing mycobacterium. A plethora of intrinsic resistance mechanisms renders almost all clinically used antibiotics ineffective against *M. abscessus* (Nessar et al., 2012). In addition, the presence of a highly dynamic open pan-genome in *M. abscessus* might explain the ease with which the bacterium evolves and adapts to a wide-spectrum of stressful environmental conditions encountered in diverse habitats (Choo et al., 2014; Wu et al., 2018). Importantly, the respiratory habitat of *M. abscessus* brings it in close proximity to highly virulent pathogens (for example, *Pseudomonas aeruginosa* in cystic fibrosis lung) which can serve as donors of novel drug resistance or virulence genes (Ripoll et al., 2009).

Treatment of an *M. abscessus* pulmonary infection is very difficult owing to the bacterium’s high-level innate resistance towards most antibiotics commonly used for Gram-negative and Gram-positive bacterial infections including majority of β-lactams, tetracyclines, aminoglycosides and macrolides. In addition, anti-TB drugs including first-line drugs (such as rifampicin and isoniazid) as well as some second-line agents (such as capreomycin) are ineffective against this pathogen (**Table 1**). Thus, the term “incurable nightmare” is often used to describe *M. abscessus* (Brown-Elliott et al., 2012; Nessar et al., 2012). So far, no reliable antibiotic regimen has been established for *M. abscessus* pulmonary disease. Antibiotic administration is largely empirical and relies on *in vitro* antibiotic susceptibility testing and definitive subspecies identification (Griffith et al., 2007; Ryu et al., 2016). Treatment of *M. abscessus* pulmonary disease as recommended by the British Thoracic Society involves administration of a multi-drug regimen encompassing intravenous antibiotics - amikacin, tigecycline, and imipenem along with an oral macrolide (clarithromycin or azithromycin) for clinical isolates susceptible to macrolides, during the initial treatment phase. For the continuation phase of treatment, nebulised amikacin and an oral macrolide combined with one to three of the following oral antibiotics: linezolid, clofazimine, minocycline, co-trimoxazole, and moxifloxacin are generally recommended (Haworth et al., 2017). Standard of care calls for continuation of antibiotic therapy for a minimum of 12 months after culture conversion. However, microbiological eradication of *M. abscessus* bacilli from lung tissues, using recommended antibiotic regimens, is rare and recurrence is often after completion of a treatment course and successful symptom management (Jarand et al., 2011; Stout et al., 2016).

**Table 1:** Drug susceptibility of *Mycobacterium abscessus* wild-type and isogenic mutants in comparison to screening concentrations for *M. tuberculosis* with decreased susceptibility.

Antimicrobial agent	*M. tuberculosis*^∗^ (mg/L)	*M. abscessus* MIC (mg/L)	*M. abscessus* mutant MIC (mg/L)	Reference
Isoniazid	0.1	>512	*katG^+^ (M. tb)*: 32	Pers. Communication
Rifampicin	1.0	128	Δ*arr*: 0.25	Rominski et al., 2017a
Ethambutol	5.0	64 (polymorphism in target gene)	N.A.	Alcaide et al., 1997
Capreomycin	2.5	>256	Δ*eis2*: 4	Rominski et al., 2017c
Clarithromycin	N.D.	64	Δ*erm*: 0.5	Choi et al., 2012
Kanamycin B	N.D.	8	Δ*aac(2*′*)*: 0.125	Rominski et al., 2017c
Amikacin	1.0	4	Δ*eis2*: 0.25	Rominski et al., 2017c
Streptomycin	1.0	32	Δ*str(3*″*)*: 2	Dal Molin et al., 2017
Tetracycline	N.D.	60	Δ*tetX*: 4	Rudra et al., 2018
Amoxicillin	N.D.	>256	Δ*bla*: 8	Dubée et al., 2015
Ampicillin	N.D.	>256	Δ*bla*: 4	Dubée et al., 2015


*Epidemiologic cutoff values (ECOFF; MGIT960) as defined by Cambau et al. (2015); MIC, minimal inhibitory concentration; N.A., Not available; N.D., Not determined.*

As a result of extensive, repeated or inappropriate use of macrolides and aminoglycosides, which inhibit protein biosynthesis by binding to the large and small ribosomal subunits, respectively, *M. abscessus* strains with clinically acquired pan-macrolide and pan-aminoglycoside resistance have emerged, due to mutation(s) in the corresponding 23S (*rrl*) and 16S (*rrs*) rRNA genes (Wallace et al., 1996; Prammananan et al., 1998; Maurer et al., 2012; Nessar et al., 2012). Acquired resistance to macrolides and aminoglycosides severely limits the remaining treatment options and highlights the urgent need for new antimicrobial agents.

**FIGURE 1:**
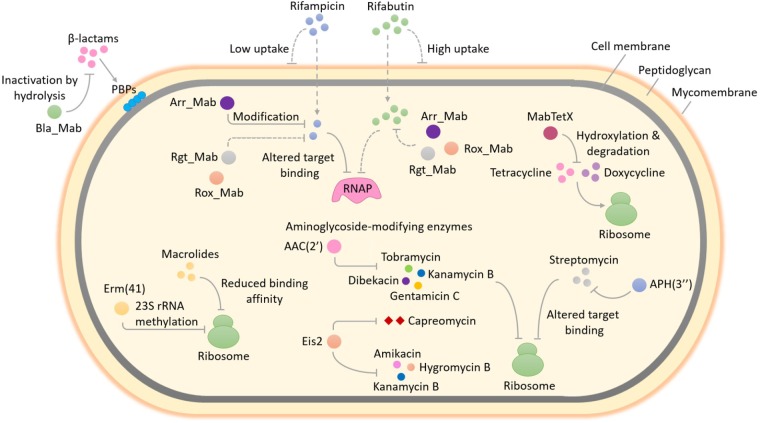
Enzyme-mediated antibiotic resistance in *Mycobacterium abscessus. M. abscessus* is intrinsically resistant to a large and diverse array of antimicrobial agents. The occurrence of several enzymes that can modify and/or degrade antibiotics or alter their targets in *M. abscessus* enables it to resist the action of multiple classes of antibiotics. This figure provides an overview of the well-studied as well as some unexplored enzyme-mediated resistance mechanisms in *M. abscessus.* Methylation of 23S rRNA by Erm(41), an erythromycin ribosome methylase, lowers the binding affinity of macrolides for the ribosome exit tunnel and confers macrolide resistance. The expression of *erm*(41) gene in *M. abscessus* is induced upon exposure to macrolides, therefore this type of resistance by target modification is known as inducible macrolide resistance. The addition of different chemical groups including acyl and phosphate groups to vulnerable sites on the aminoglycoside molecule by *M. abscessus* enzymes can prevent the binding of aminoglycoside to its ribosomal target due to steric hindrance and result in resistance. A diverse group of enzymes that differ in the aminoglycosides that they can modify as well as in the region of the antibiotic that is modified, are found in *M. abscessus*. These include two putative acetyltransferases - AAC(2′) and Eis2 and one putative phosphotransferase - APH(3″). The substrate aminoglycosides for each enzyme are shown. In addition to aminoglycosides, Eis2 can also modify other ribosome-targeting antibiotics like capreomycin. Resistance to rifampicin and other rifamycin antibiotics in *M. abscessus* involves covalent modification and drug inactivation by an ADP-ribosyltransferase, Arr_Mab. Apart from ADP-ribosylation, two additional group transfer mechanisms of rifampicin inactivation including glycosylation, phosphorylation as well as decomposition of rifampicin by monooxygenation, are widespread in environmental bacteria but have not been explored in mycobacteria. Being a saprophyte in soil, exposure to antibiotic producing actinomycetes that utilize a variety of rifamycin inactivating mechanisms in its natural habitat might have favored the selection of an unknown reservoir of rifamycin resistance genes in *M. abscessus*, for example, those encoding a rifamycin glycosyltransferase (Rgt_Mab) and/or a rifamycin monooxygenase (Rox_Mab) (dashed lines). *M. abscessus* also produces a tetracycline-degrading flavin monooxygenase, MabTetX that activates molecular oxygen to hydroxylate and destabilize the antibiotic, which subsequently undergoes non-enzymatic decomposition, thereby conferring resistance. Resistance to β-lactams in *M. abscessus* is afforded by the production of a hydrolytic β-lactamase enzyme, Bla_Mab with the ability to degrade a broad range of β-lactams including extended-spectrum cephalosporins and carbapenems. RNAP, RNA polymerase; PBPs, penicillin-binding proteins; AAC(2′), 2′-*N*-acetyltransferase; Eis2, enhanced intracellular survival protein 2; APH(3″), aminoglycoside 3″-*O*-phosphotransferase.

Mechanisms underpinning intrinsic drug resistance of *M. abscessus* are multi-fold and fall into two main groups: first, the presence of a highly impermeable cell envelope and/or multi-drug efflux pumps might reduce the effective concentration of antibiotics within the bacterial cells; second, the genome of *M. abscessus* encodes several putative enzymes which can inactivate antibiotics by modification and/or degradation or lower the affinity of the drug for its target by modifying the target (Ripoll et al., 2009; Nessar et al., 2012). For long, molecular investigations aimed at elucidating antibiotic resistance mechanisms of *M. abscessus* were limited, however, significant progress has been made in recent years owing to the development of efficient tools for genetic manipulation of this bacterium (**Figure 1**). In this article, we provide an up-to-date overview of the main molecular mechanisms of antibiotic resistance in *M. abscessus* with a focus on the drug-target-modifying or drug-modifying enzymes and discuss the potential impact on clinical treatment. We also discuss how this knowledge could facilitate the discovery and development of new improved antimicrobial agents or help rescue the function of currently available antibiotics against *M. abscessus* and provide an update on the latest research underway to combat multi-drug resistant *M. abscessus* infections.

## Antibiotic-Target-Modifying Enzymes in *M. Abscessus*

### Macrolide Resistance: MAB_2297 [*erm*(41)] Encoded Erythromycin Ribosome Methylase

Macrolides are among the most successful antibiotics in the world and are highly prescribed for infections caused by non-tuberculous mycobacteria. A multidrug regimen recommended for *M. abscessus* infections almost always includes macrolides, particularly clarithromycin or azithromycin. Macrolides target the nascent polypeptide exit tunnel within the 50S ribosomal subunit, which accommodates the newly synthesized polypeptide chain as it emerges from the ribosome. Following macrolide binding, the elongation of polypeptide chain continues up to a few amino acids before the peptidyl-tRNA dissociates from the ribosome thereby arresting protein synthesis (Wilson, 2014).

In bacteria, innate resistance towards macrolides is most often the result of increased efflux, ribosomal methyltransferases and GTP-dependent macrolide kinases (Pawlowski et al., 2018). A well-studied example of macrolide resistance by target modification in bacteria is the erythromycin ribosome methylase (Erm) enzyme, which specifically methylates A2058 nucleotide of 23S rRNA and lowers the macrolide affinity for ribosome exit tunnel (Blair et al., 2015). An Erm methyltransferase is also responsible for inducible macrolide resistance in *M. abscessus* (**Figure 1**). Interestingly, the *erm*(41) gene, first described by Nash et al. (2009), is only functional in two out of three subspecies of the *M. abscessus* complex. The subspecies *M. massiliense* harbors deletions in its *erm*(41) gene copy which render the Erm(41) enzyme inactive and the bacterium susceptible to macrolides. This may explain why patients with *M. massiliense* infections have better treatment outcomes than patients with *M. abscessus* or *M. bolletii* infections. To further complicate matters, a T/C polymorphism at position 28 of the *erm*(41) sequence determines the appearance of inducible macrolide resistance in *M. bolletii* and *M. abscessus* subspecies. Only those isolates that harbor a T28 *erm*(41) sequevar develop inducible macrolide resistance. Phenotypic detection of inducible macrolide resistance in the *M. abscessus* complex by drug susceptibility testing requires extended incubation (14 days rather than 3 days) in the presence of the drug (Nash et al., 2009; Kim et al., 2010).

A functional role of *erm*(41) in inducible resistance to macrolides has been confirmed by genetic studies. Deletion of *erm*(41) in *M. abscessus* lowered the minimal inhibitory concentration (MIC) of clarithromycin by 128-fold (**Table 1**), while introduction of a functional *M. abscessus erm*(41) gene into *M. massiliense* led to a 64-fold increase in the clarithromycin MIC on day 14 (Choi et al., 2012). Inducible Erm(41)-dependent rRNA methylation severely affects the efficacy of macrolides against *M. abscessus.* The long duration of partially effective macrolide therapy, eventually favors the emergence of isolates with high-level constitutive macrolide resistance. Mutations that alter the 23S rRNA nucleotides 2058 and 2059 within the peptidyl-transferase center (PTC), are a frequent cause of constitutive macrolide resistance in *M. abscessus* (Wallace et al., 1996; Bastian et al., 2011; Brown-Elliott et al., 2015).

## Antibiotic-Modifying Enzymes in *M. Abscessus*

### Aminoglycoside Resistance: MAB_4395 [*aac(2*′*)*] & MAB_4532c (*eis2*) Encoded Acetyltransferases; MAB_2385 Encoded 3″-*O*-Phosphotransferase

Aminoglycosides are among the broadest classes of antibiotics that interfere with specific steps in bacterial protein synthesis (Kohanski et al., 2010). All aminoglycosides primarily target the 16S rRNA component of 30S ribosomal subunit, however, their exact binding site and mode of action may differ slightly depending on their chemical structure. For example, 2-deoxystreptamine (2-DOS) aminoglycosides (such as kanamycin, amikacin, apramycin, arbekacin), which are chemically distinct from the non-DOS aminoglycosides (for example, streptomycin), specifically bind to the A-site region of small ribosomal subunit at the decoding center, encompassing conserved nucleotides A1492 and A1493 of 16S rRNA. 2-DOS aminoglycosides function by inhibiting the translocation of tRNA-mRNA complex through the ribosome. Additionally, several members of this class also induce protein mistranslation by promoting and stabilizing the interaction of non-cognate aminoacylated tRNAs with mRNA. In contrast, the non-DOS aminoglycoside streptomycin targets a different region of 16S rRNA in proximity of the decoding center and mainly interferes with the delivery of aminoacylated tRNA to the A-site (Wilson, 2014).

Bacterial resistance to aminoglycosides may occur due to low cell permeability/efflux, mutations or modification of 16S rRNA, mutations in ribosomal protein genes and enzymatic drug modification. Drug and target modification being the most extensively studied and clinically relevant aminoglycoside resistance mechanisms in pathogenic bacteria. Covalent modification of key amino or hydroxyl groups within the aminoglycoside molecule by bacterial enzymes markedly decreases the binding affinity for the ribosomal target and confers aminoglycoside resistance. Aminoglycoside-modifying enzymes in bacteria can be grouped into three main classes namely, acetyltransferases, nucleotidyltransferases, and phosphotransferases (D’Costa et al., 2011; Blair et al., 2015). A worrying discovery that the genome of emerging pathogen *M. abscessus* encodes several putative aminoglycoside-modifying enzymes which include members of all three classes, came in 2009 when Ripoll et al first sequenced the complete genome of *M. abscessus* (Ripoll et al., 2009). Some predictions were later substantiated by drug susceptibility testing and biochemical analysis (Maurer et al., 2015). More recently, direct genetic evidence for a functional role of some of these putative aminoglycoside-modifying enzymes in *M. abscessus* intrinsic resistance to this antibiotic class, was obtained.

Similar to all other mycobacteria that have been studied so far (Aínsa et al., 1997), *M. abscessus* also harbors a 2′-*N*-acetyltransferase [AAC(2′)], encoded by MAB_4395 gene. This enzyme is capable of acetylating several aminoglycosides bearing a 2′ amino group including gentamicin C, dibekacin, tobramycin and kanamycin B (**Figure 1**). This was evident upon deletion of MAB_4395 in *M. abscessus* ATCC 19977 strain which led to a 4-64-fold reduction in the MICs of these aminoglycosides (**Table 1**) (Rominski et al., 2017c). Interestingly, another *N-*acetyltransferase capable of conferring aminoglycoside resistance in *M. abscessus* is a homolog of *Anabaena variabilis* enhanced intracellular survival (Eis) protein (**Figure 1**). Eis proteins are widespread in mycobacteria as well as other Gram-positive bacteria. Eis protein in *Mycobacterium tuberculosis* has been shown to enhance intracellular survival within macrophages by acetylating and activating dual-specificity protein phosphatase 16/mitogen-activated protein kinase phosphatase-7 (DUSP16/MPK-7), a JNK-specific phosphatase that inhibits inflammation, autophagy and subsequent death of infected macrophages (Shin et al., 2010; Kim et al., 2012). Furthermore, upregulation of *eis* gene expression owing to mutations in its promoter was associated with kanamycin resistance in one-third of clinical isolates encompassing a large collection of *M. tuberculosis* strains from diverse geographical locations (Zaunbrecher et al., 2009). More recently, structural and biochemical characterization of *M. tuberculosis* Eis revealed its unprecedented ability to acetylate multiple amines of several aminoglycosides (Chen et al., 2011).

Analysis of *M. abscessus* genome revealed two genes namely, MAB_4124 (*eis1*) and MAB_4532c (*eis2*), which show homology to *eis* from *M. tuberculosis* (Rv2416c) and *A.variabilis* (Ava_4977), respectively. Drug susceptibility testing could confirm a functional role for Eis2 but not Eis1, in intrinsic aminoglycoside resistance. Of note, deletion of MAB_4532c enhanced *M. abscessus* susceptibility towards a heterogenous group of aminoglycosides including the cornerstone drug amikacin as well as a non-aminoglycoside antibiotic capreomycin (**Table 1**) (Rominski et al., 2017c). Furthermore, heterologous expression of *eis2* in *Mycobacterium smegmatis* decreased amikacin susceptibility (Hurst-Hess et al., 2017). Capreomycin is a cyclic peptide antibiotic that inhibits protein synthesis by blocking peptidyl-tRNA translocation. The drug is particularly active against *M. tuberculosis* and commonly used as a second-line agent for the treatment of TB (Stanley et al., 2010; Wilson, 2014). Although remarkable, the discovery that *M. abscessus* Eis2 exhibits a broad-spectrum acetyltransferase activity is not a real surprise. In fact, recent structural and functional studies on Eis enzymes from *M. tuberculosis* (Eis_*Mtb*), *M. smegmatis* (Eis_*Msm*), and *A. variabilis* (Eis_*Ava*) revealed the presence of an unusually large and complex active site unlike in other aminoglycoside acetyltransferases. The unique structure equips Eis with a regio-versatile multi-acetylating acetyltransferase activity towards a broad range of substrates (Chen et al., 2012; Pricer et al., 2012; Jennings et al., 2013). However, there seems to be a notable difference between the acetylation activities of *M. abscessus* Eis2 and *M. tuberculosis* Eis especially towards capreomycin as the MIC of capreomycin in *M. abscessus* is about 100-fold higher than that in *M. tuberculosis* and this difference is almost completely abolished by the deletion of *eis2* gene (**Table 1**) (Rominski et al., 2017c).

Aminoglycoside acetyltransferases Eis2 and AAC(2′) do not appear to modify streptomycin, an aminoglycoside which exhibits moderate *in vitro* activity against *M. abscessus* (MIC: 32 mg/L) (Rominski et al., 2017c). Instead, a MAB_2385 encoded putative aminoglycoside 3″-*O*-phosphotransferase is the main determinant for intrinsic streptomycin resistance in *M. abscessus* (**Figure 1**). Twelve putative aminoglycoside phosphotransferases are encoded in the genome of *M. abscessus* (Ripoll et al., 2009). Among them, MAB_2385 was found to be a close homolog of *aph(3*″*)-Ic*, an aminoglycoside 3″-*O*-phosphotransferase gene which was previously shown to confer streptomycin resistance in *Mycobacterium fortuitum* (Ramón-García et al., 2006). Deletion of MAB_2385 enhanced the susceptibility of *M. abscessus* towards streptomycin by 16-fold (**Table 1**). Furthermore, MAB_2385 was able to confer streptomycin resistance when heterologously expressed in *M. smegmatis* (Dal Molin et al., 2017). To the best of our knowledge other annotated aminoglycoside phosphotransferases (such as MAB_0163c, MAB_0313c, MAB_0327, MAB_0951, and MAB_1020) and aminoglycoside acetyltransferases (for example, MAB_0247c, MAB_0404c, MAB_0745, MAB_4235c, and MAB_4324c) have not been addressed experimentally, however, interesting regulatory features of drug resistance genes have been elucidated.

A WhiB7 like protein encoded by MAB_3508c was recently uncovered as a multi-drug inducible transcriptional regulator that modulates the expression of genes conferring aminoglycoside and macrolide resistance in *M. abscessus*. WhiB7, an autoregulatory transcriptional activator, is a determinant of innate antibiotic resistance in mycobacteria. In *M. tuberculosis*, genes conferring antibiotic resistance including *ermMT* (Rv1988), *eis* (Rv2416c) and *tap* (Rv1258c) are induced in a *whiB7*-dependent manner, upon exposure to antibiotics. Similarly, the *erm*(41) and *eis2* genes in *M. abscessus* are included in the *whiB7* regulon (Hurst-Hess et al., 2017). A recent study by Pryjma et al. (2017) demonstrated that exposure to sub-inhibitory levels of clarithromycin, enhanced amikacin as well as clarithromycin resistance in *M. abscessus* in a *whiB7*-dependent manner. Of note, deletion of MAB_3508c rendered *M. abscessus* more susceptible to amikacin (fourfold) and clarithromycin (eightfold), while complementation with an intact gene copy restored resistance to these antibiotics, suggesting a role of *whiB7* in *M. abscessus* amikacin and clarithromycin resistance. Interestingly, pre-exposure to clarithromycin, which is a potent inducer of *M. abscessus whiB7*, enhanced the resistance of *M. abscessus* wild-type strain towards amikacin by fourfold but had no effect on amikacin susceptibility of a ΔwhiB7 mutant. In addition, pre-treatment with clarithromycin markedly increased resistance to itself in the wild-type strain but did not alter clarithromycin sensitivity of a Δ*whiB7* mutant. Furthermore, a quantitative reverse transcription-PCR assay revealed a *whiB7*-dependent upregulation of *eis2* and *erm*(41) genes following pre-exposure to clarithromycin. These observations support the following conclusions: (i) in *M. abscessus*, *whiB7* (MAB_3508c) activates the expression of genes which confer amikacin and clarithromycin resistance, i.e., *eis2* and *erm*(41), (ii) strong induction of *whiB7* following exposure to clarithromycin confers cross-resistance to amikacin in addition to activating inducible macrolide resistance (Pryjma et al., 2017). Thus, *in vitro* findings by Pryjma et al. (2017) suggest that front-line antibiotics, amikacin and clarithromycin may exhibit antagonistic effects when used in combination for the treatment of *M. abscessus* infections. This may at least partially explain the limited clinical efficacy of currently recommended multi-drug regimen for *M. abscessus* which almost always includes these two antibiotics.

### Tetracycline Resistance: MAB_1496c Encoded Flavin Monooxygenase

Tetracyclines are a class of broad-spectrum natural product antibiotics that interfere with bacterial protein synthesis. These drugs bind with high affinity to the small ribosomal subunit and specifically interfere with the delivery of aminoacylated tRNA to the A-site (Kohanski et al., 2010; Wilson, 2014). While tetracyclines are an important class of ribosome-targeting antibiotics, their anthropogenic and prolific use in the clinic and agriculture has led to emergence of resistance among benign as well as pathogenic bacteria, thereby limiting their efficacy. Despite growing resistance, tetracyclines continue to remain one of the most successful and widely used chemotherapeutics against bacterial infections (Allen et al., 2010). Furthermore, a renaissance for tetracyclines is being fuelled by the development of next-generation derivatives such as tigecycline, approved for clinical use in 2005 and omadacycline and eravacycline, undergoing late-phase clinical trials (Kinch and Patridge, 2014). In fact, tigecycline is the only new antibiotic and the sole member of its class, which has been introduced in the treatment of recalcitrant *M. abscessus* lung disease with clinical evidence (Wallace et al., 2014).

Previously, resistance towards tetracyclines was thought to be exclusively mediated by two mechanisms namely, drug efflux and ribosome protection. However, evidence for enzymatic inactivation of tetracyclines by a family of flavin adenine dinucleotide (FAD)-dependent monooxygenases in both benign and pathogenic bacteria has been recently documented. Few well characterized examples of such tetracycline-modifying enzymes include TetX from the obligate anaerobe *Bacteroides fragilis* and Tet(56) found in human pathogen *Legionella longbeachae*, the causative agent of Legionnaires’ disease and Pontiac fever (Yang et al., 2004; Park et al., 2017). Whether such a mechanism of tetracycline resistance existed in mycobacteria was not known until recently, when Rudra and colleagues demonstrated through elegant experiments, that high levels of intrinsic resistance towards tetracycline and second generation derivative doxycycline in *M. abscessus* was mediated by a flavin monooxygenase, MabTetX (**Figure 1**). Deletion of MAB_1496c (encoding a putative FAD binding monooxygenase) enhanced the susceptibility of *M. abscessus* towards tetracycline and doxycycline by 15–20-fold (**Table 1**), while a complemented ΔMAB_1496c strain exhibited even higher levels of resistance towards both antibiotics compared to the wild-type strain, supporting the notion that MAB_1496c is a primary determinant for *M. abscessus* intrinsic tetracycline resistance. Furthermore, UV-Visible and mass spectrometry assays with purified MAB_1496c protein provided evidence for hydroxylation and subsequent degradation of tetracycline and doxycycline by MabTetX (Rudra et al., 2018). Interestingly, MAB_1496c is not a member of the *whiB7* regulon, even though tetracycline is a strong inducer of *whiB7* in *M. abscessus* (Hurst-Hess et al., 2017). In fact, MabTetX expression is regulated by a TetR family repressor, MabTetR_x_ (encoded by the upstream gene MAB_1497c) which represses the MAB_1497c-MAB_1496c operon by binding to a 35bp operator sequence *tetO*. This repression is relieved in the presence of tetracycline and doxycycline which can bind the repressor protein thereby preventing its interaction with the *tetO* operator (Rudra et al., 2018).

The glycylcycline antibiotic tigecycline, specifically designed to evade resistance by ribosome protection and efflux, is not invulnerable to modification by *Bacteroides* TetX, which was recently also identified in numerous clinically relevant pathogens (Volkers et al., 2011; Leski et al., 2013). Although tigecycline was found to be a poor substrate of TetX, alarmingly enzymatic activity of TetX was substantially improved upon acquisition of single amino acid substitutions to confer resistance at clinically relevant tigecycline concentrations when overexpressed in *Escherichia coli* (Linkevicius et al., 2016). Surprisingly, tigecycline resisted MabTetX-dependent degradation and did not induce its expression, which might contribute to its excellent *in vitro* activity and moderate clinical efficacy against *M. abscessus* (Rudra et al., 2018). However, selective pressure exerted by tigecycline will eventually favor the emergence of *M. abscessus* isolates carrying extended-spectrum FAD-monooxygenases with the ability to modify glycylcyclines.

Another interesting feature is the ability of anhydrotetracycline (ATc), a degradation product of tetracycline with poor antibiotic activity which was recently shown to be a competitive inhibitor of TetX and Tet(56) flavin monooxygenases, to rescue antibiotic activities of tetracycline and doxycycline against *M. abscessus* expressing MabTetX (Park et al., 2017; Rudra et al., 2018). However, ATc was also found to induce MabTetX expression by binding the MabTetR_x_ repressor, a property at odds with its MabTetX inhibition activity. While this finding together with the severe side effects associated with ATc use, makes it an unattractive drug candidate, it nevertheless represents a flexible starting point for developing MabTetX inhibitors with improved activity, better tolerability and minimal binding affinity towards MabTetR_x_. Co-administration with a potent MabTetX monooxygenase inhibitor can potentially rescue the clinical efficacies of tetracycline and doxycycline which are currently inactive against the *M. abscessus* complex (Burgos et al., 2011; Rudra et al., 2018).

### β-Lactam Resistance: MAB_2875 Encoded β-Lactamase

β-lactams constitute an important class of antibiotics that function by inhibiting cell wall synthesis in bacteria. The β-lactam structure closely resembles the terminal D-alanyl-D-alanine dipeptide of peptidoglycan, a substrate of penicillin binding proteins (also known as D, D-transpeptidases) that catalyze the final cross-linking step of peptidoglycan synthesis. Penicilloylation of the D, D-transpeptidase active site following treatment with β-lactams, disables the enzyme and prevents the formation of peptidoglycan cross-links, thereby interfering with cell wall biosynthesis (Kohanski et al., 2010). Despite being the cornerstones of antimicrobial chemotherapy and constituting one of the largest groups of antibiotics available today, only two members of the β-lactam class namely, cefoxitin (a cephalosporin) and imipenem (a carbapenem) form a part of the antibiotic arsenal for *M. abscessus*. Furthermore, cefoxitin and imipenem have been shown to exhibit only moderate *in vitro* activity against *M. abscessus* with MICs of 32 and 4 mg/L, respectively (Lavollay et al., 2014). Majority of the other β-lactam antibiotics are not effective against this bacterium.

Several possible mechanisms may contribute to β-lactam resistance in *M. abscessus.* Low mycomembrane permeability and β-lactamase production may reduce the effective concentration of β-lactams at the site of action. Alternatively, modification of transpeptidase profile of the cell by replacement of penicillin binding proteins (D, D-transpeptidases) with L, D-transpeptidases for instance, may reduce the activity of penicillins and cephalosporins that have lower affinity for L, D-transpeptidase (Wivagg et al., 2014). However, most of the abovementioned resistance mechanisms are relatively unexplored in this organism except for one. Recently it was shown that the genome of *M. abscessus* encodes a strong, constitutive class A β-lactamase (Bla_Mab) which renders it highly resistant towards most β-lactams (**Figure 1**). The *M. abscessus* β-lactamase Bla_Mab, encoded by MAB_2875 is endowed with an exceptional broad-spectrum activity and can effectively hydrolyse several members of first- and second-generation cephalosporins, carbapenems, and penams (Soroka et al., 2014). The currently recommended β-lactams namely imipenem and cefoxitin are substrates of Bla_Mab, however, they are hydrolysed at a very slow rate, which may contribute to their clinical efficacy. The instability of imipenem complicates drug susceptibility testing when generation times of test bacteria require incubations for several days as in the case of *M. abscessus* (Rominski et al., 2017b).

One strategy to overcome the hurdle of chromosomally encoded β-lactamase in *M. abscessus* is to co-administer a β-lactamase inhibitor with the failing β-lactam. Soroka and colleagues evaluated the *in vitro* efficacy of nitrocefin and meropenem in combination with approved β-lactamase inhibitors clavulanate, tazobactam, and sulbactam against Bla_Mab. Surprisingly, Bla_Mab inhibition was not detected for all the three inhibitor drugs (Soroka et al., 2014). This remarkable ability of Bla_Mab to resist inhibition even surpasses that of BlaC, a class A β-lactamase encoded by its relative *M. tuberculosis*. Although BlaC is able to reverse inhibition caused by tazobactam and sulbactam, and return to its native functional form, clavulanate can slowly but irreversibly inhibit this enzyme (Sagar et al., 2017). In fact, a recent demonstration of *in vitro* synergy between meropenem and clavulanate in 13 extensively drug resistant (XDR) *M. tuberculosis* strains raised a renewed interest in β-lactams for use in TB therapy (Hugonnet et al., 2009). Fortunately, a surprising finding that *M. abscessus* Bla_Mab is effectively disabled by the newly approved β-lactamase inhibitor avibactam provides a new hope for old antibiotics. A study by Dubée et al. (2015) showed that co-administering a small amount of avibactam (4 mg/L) with each of several representative antibiotics from three important β-lactam sub-classes (carbapenems, penams, and cephalosporins) lowered their MICs in *M. abscessus* CIP104536 strain to levels comparable to those observed in a β-lactamase deficient mutant. The authors also reported synergy between avibactam and amoxicillin within macrophages as well as in a zebrafish model of *M. abscessus* infection (Dubée et al., 2015). More recently, an *in vitro* synergy screen of 110 β-lactam - β-lactamase inhibitor combinations against an *M. abscessus* clinical isolate identified six potential hits. Five selected β-lactamase inhibitors encompassing both β-lactam-based (clavulanate, tazobactam, and sulbactam) as well as non-β-lactam-based inhibitors (avibactam and vaborbactam) were evaluated for synergistic effects with a large panel of β-lactams. Importantly, all observed synergy combinations exclusively involved non-β-lactam-based inhibitors, either avibactam or vaborbactam. Combinations of avibactam with each of the following drugs including ampicillin, amoxicillin, tebipenem, and panipenem displayed synergistic effects against *M. abscessus.* Furthermore, the carbapenems, tebipenem, and panipenem also exhibited synergy with a novel boronic acid β-lactamase inhibitor, vaborbactam (Aziz et al., 2018). Thus, non-β-lactam-based β-lactamase inhibitors such as avibactam and vaborbactam can potentially extend the spectrum of β-lactams employed for the treatment of largely incurable, chronic *M. abscessus* pulmonary infections.

### Rifamycin Resistance: MAB_0591 Encoded ADP-Ribosyltransferase

Rifampicin, a rifamycin antibiotic commonly employed as a first-line agent in the treatment of *M. tuberculosis* infections, hardly exhibits any activity towards the *M. abscessus* complex (MIC: 128 mg/L) (**Table 1**). Since rifamycins inhibit transcription by binding with high affinity to *rpoB* encoded β-subunit of bacterial RNA polymerase, resistance towards this group of antibiotics is most commonly conferred by point mutations within the target gene *rpoB*, especially in *M. tuberculosis* (Campbell et al., 2001). However, additional rifamycin resistance mechanisms that involve enzymatic drug inactivation are also prevalent in other mycobacterial species. The presence of a rifamycin ADP-ribosyltransferase in *M. smegmatis* was known for quite a while (Quan et al., 1997), however, only recently genetic studies involving gene knockout and heterologous expression unveiled the existence of a MAB_0591 encoded ADP-ribosyltransferase as the major determinant for high levels of innate rifamycin resistance in *M. abscessus* (**Figure 1**). Deletion of MAB_0591 enhanced the susceptibility of *M. abscessus* towards three tested rifamycins including rifaximin, rifapentine and rifampicin by 64–512-fold, respectively (Rominski et al., 2017a).

Surprisingly, a rifampicin derivative, rifabutin showed up as an attractive hit (MIC: 2.5 mg/L), when a set of 2,700 FDA-approved drugs were screened against a clinical isolate of *M. abscessus.* Rifabutin exhibited approximately 10-fold higher potency *in vitro* relative to rifampicin and rifapentine, against reference strains representative of all three subspecies of the *M. abscessus* complex as well as a collection of clinical isolates and its bactericidal activity was comparable to or better than that of clarithromycin, one of the cornerstone drugs for *M. abscessus* infections (Aziz et al., 2017). The higher efficacy of rifabutin relative to rifampicin in *M. abscessus* as well as in *M. tuberculosis* (with no known rifamycin inactivating enzymes) suggests its increased accumulation within the bacterial cells. This may be due to differences in membrane penetration or specificity of efflux systems towards different rifamycins (**Figure 1**). Based on the findings reported by Aziz et al. (2017), rifampicin analog rifabutin, appears to be a potential candidate for repurposing and its clinical efficacy in patients with *M. abscessus* pulmonary disease should be further explored.

## Clinical Relevance

Although the inherent ability of *M. abscessus* to exhibit resistance to a wide array of antibiotics has long been recognized, our knowledge of the prodigious diversity of mechanisms involved has improved immensely in recent years, owing to the development of tools for genetic manipulation of *M. abscessus*. A better understanding of the genetic basis of innate antibiotic resistance is a prerequisite for the discovery and development of synergistic drug combinations, where one agent serves to salvage the antibacterial activity of a failing antibiotic by inhibiting an intrinsic resistance mechanism. This is exemplified by the recent discovery that avibactam is a potent inhibitor of *M. abscessus* β-lactamase Bla_Mab. When used in combination, avibactam lowered the MICs of several β-lactams in *M. abscessus* by 4–32-fold (Dubée et al., 2015; Kaushik et al., 2017). Despite the success of β-lactamase inhibitors, the concept of designing drugs which target intrinsic resistance mechanisms in bacteria, has not gained substantial clinical exploitation beyond this antibiotic class (Brown, 2015). Recently, a repertoire of drug-modifying and target-modifying enzymes conferring innate resistance to aminoglycosides, tetracyclines, rifamycins, and macrolides in *M. abscessus* were delineated (Nash et al., 2009; Dal Molin et al., 2017; Rominski et al., 2017a,c; Rudra et al., 2018). Likewise, attempts to identify chemical entities which could potentially rescue the function of one or more key members from each aforementioned antibiotic group, should be pursued in the near future.

Since the discovery that mutations within *eis* promoter are associated with kanamycin resistance in *M. tuberculosis*, vigorous attempts to identify Eis inhibitors that can potentially rescue kanamycin activity in this pathogen have been pursued with notable success. Recently, several structurally diverse competitive Eis inhibitors with 1,2,4-triazino[5,6*b*]indole-3-thioether-based, sulfonamide-based, pyrrolo[1,5-*a*]pyrazine-based and isothiazole *S,S*-dioxide heterocyclic scaffolds were identified by high-throughput screenings of large compound libraries. Importantly, few promising inhibitors could completely restore kanamycin susceptibility in a kanamycin resistant *M. tuberculosis* strain *in vitro* at concentrations that were non-cytotoxic to mammalian cells (Green et al., 2012; Garzan et al., 2016, 2017; Willby et al., 2016; Ngo et al., 2018). Remarkably, inhibitors designed for *M. tuberculosis* Eis, were also found to be active against Eis homologs in *M. smegmatis* and *A. variabilis* (Chen et al., 2012; Pricer et al., 2012). These findings give rise to an intriguing possibility that Eis_*Mtb* inhibitors may be able to overcome Eis2 mediated resistance towards aminoglycoside and/or non-aminoglycoside antibiotics like capreomycin and restore their effectiveness against *M. abscessus.* Hence efforts to explore the activity of Eis_*Mtb* inhibitors against *M. abscessus* Eis2 enzyme should be undertaken. Alternatively, extending drug discovery efforts towards targeting WhiB7, could be a possible strategy to tackle intrinsic macrolide and aminoglycoside resistance in *M. abscessus.* WhiB7 is a conserved transcriptional regulator in mycobacteria that co-ordinates intrinsic resistance to a wide range of ribosome-targeting drugs (Burian et al., 2012). Recent evidence suggests that genes which contribute to intrinsic aminoglycoside and macrolide resistance in *M. abscessus*, i.e., *eis2* and *erm*(41), are induced in a *whiB7*-dependent manner upon exposure to ribosomal antibiotics (Hurst-Hess et al., 2017). Thus, with the aim to kill two birds with one stone, compounds that prevent the induction of *eis2* and *erm*(41) resistance genes in *M. abscessus* by modulating the synthesis and/or activity of WhiB7, should be sought.

Since the discovery and development of compounds that can circumvent existing resistance mechanisms in *M. abscessus* is a daunting and time-consuming task, an immediate and relatively simple solution to improve treatment efficacy may involve repurposing and repositioning of currently available antibiotics. A number of candidate drugs suited for this purpose have been reviewed in detail elsewhere (Wu et al., 2018) and are therefore only briefly mentioned here. For example, a systematic screening of a collection of FDA-approved drugs revealed rifabutin, an analog of rifampicin, exhibiting potent *in vitro* activity against *M. abscessus* (MIC: 2.5 mg/L) (Aziz et al., 2017). Similarly, clofazimine, a leprosy drug currently repurposed for treatment of TB, was also found to be active against *M. abscessus.* In addition to promising *in vitro* activity (MIC ≤ 1 mg/L), clofazimine displayed adequate clinical efficacy and safety when evaluated in patients with *M. abscessus* pulmonary infections (Yang et al., 2017). Likewise, a novel oxazolidinone LCB01-0371, presently in Phase II clinical development for TB, exhibited high potency against *M. abscessus* compared to linezolid, an FDA-approved drug of the same class (Kim et al., 2017). Moreover, some aminoglycosides such as kanamycin A, apramycin, isepamicin, and arbekacin were also found to exhibit potent *in vitro* activity against *M. abscessus* (MICs ≤ 1 mg/L) (Rominski et al., 2017c). Besides these, other compounds that have been extensively studied for their inhibitory effects against *M. abscessus* encompass anti-TB drug bedaquiline, some TB actives like indole-2-carboxamides and piperidinol-based compound 1 (PIPD1), as well as second generation thiacetazone derivatives like D6, D15, and D17 (Dupont et al., 2016, 2017; Franz et al., 2017; Halloum et al., 2017; Kozikowski et al., 2017).

In addition, several recent studies have reported *in vitro* synergies between novel combinations of existing antibiotic classes. For example, vancomycin, an inhibitor of cell wall synthesis, exhibited synergy with clarithromycin, an inhibitor of protein synthesis, in *M. abscessus* (Mukherjee et al., 2017). Likewise, a combination of tigecycline (which inhibits protein synthesis) with teicoplanin (which disrupts peptidoglycan synthesis) displayed a strong synergistic effect against *M. abscessus* (Aziz et al., 2018). Since the mycobacterial cell envelope forms a major permeability barrier, drugs that perturb cell wall integrity, are likely to synergize with drugs having intracellular targets (Le Run et al., 2018). Such combinations based on mechanistic drug action should be further explored for synergistic effects against *M. abscessus.* For example, PIPD1 and indole-2-carboxamides, which disrupt mycolic acid synthesis by targeting MmpL3 transporter in mycobacteria (Dupont et al., 2016; Franz et al., 2017), may be able to potentiate the effects of other drugs modulating intracellular targets.

Taken together, a huge reservoir of intrinsic resistance genes combined with an unusually high evolutionary potential of its genome to acquire resistance renders almost all available antibiotics ineffective against *M. abscessus.* Therefore, in the long run, a better understanding of natural resistance mechanisms in *M. abscessus* will help jump start drug discovery projects for (i) compounds that can rescue current antibiotics by neutralizing intrinsic resistance (termed antibiotic resistance breakers), (ii) new drugs with one or more improved properties like ability to resist enzymatic modification and/or degradation, high affinity for modified or mutated targets, better solubility/uptake and low propensity for efflux (Brown, 2015). In the short run, available knowledge from studies seeking synergy between antibiotics together with those investigating potential candidates for repurposing, will aid in the development of dosing regimens that display adequate clinical efficacy and resistance to which will only emerge slowly.

## Outlook and Perspectives

Molecular genetic tools that allow investigators to systematically probe gene function in mycobacteria by generation of unmarked gene deletions and functional complementation, have proven extremely useful in dissecting the intrinsic resistome of *M. abscessus* while genetic investigations in other NTM species are still lagging behind. Using the available genetic ‘toolkit,’ 7 orthologs of known resistance gene families have been discovered so far in *M. abscessus* that provide resistance or ‘immunity’ against antibiotics targeting major cellular pathways including cell wall synthesis (β-lactams), RNA synthesis (rifamycins) and protein synthesis (macrolides, aminoglycosides, and tetracyclines). The impressive resistance diversity shown by *M. abscessus* is expected given its natural habitat is shared by many antibiotic producers, primarily the soil dwelling actinomycetes which are also reservoirs of extensively diverse resistance elements (Pawlowski et al., 2016; Crofts et al., 2017; Koteva et al., 2018). Exposure to a variety of noxious antimicrobial molecules in the soil might have favored selection of specialized and diverse resistance mechanisms in *M. abscessus* as well as in other NTM species.

Till now, the approach that researchers used to systematically probe the intrinsic resistome of *M. abscessus* involved analysis of few selected genes identified based on homology to known resistance determinants from other bacteria. As a result, this approach mainly revealed genes within *M. abscessus* that were involved in previously known resistance mechanisms and disguised the presence of potential determinants of entirely new resistance mechanisms that lack characterized homologs in other bacteria. To capture the remarkable resistance diversity of *M. abscessus* as well as other environmental NTM with unprecedented depth, future studies in this field should incorporate next-generation approaches that provide a comprehensive genome-wide definition of loci required for antibiotic resistance, using cutting-edge technologies. One such technique which can efficiently mine resistance determinants with novel sequences as well as assign resistance functions to known genes that were not previously shown to be involved in drug resistance, is the most recent incarnation of insertional mutagenesis, called transposon insertion sequencing (TIS) (Chao et al., 2016).

The utility of TIS in elucidating novel antibiotic resistance functions is highlighted by its recent application to the discovery of a repertoire of previously unknown intrinsic factors impacting resistance to antibiotics of different classes including oxazolidinones, fluoroquinolones, and aminoglycosides in pathogens of high clinical concern such as *Staphylococcus aureus*, *P. aeruginosa* and *E. coli* (Gallagher et al., 2011; Shan et al., 2015; Rajagopal et al., 2016). Screening large collections of transposon insertion mutants for antibiotic susceptibility using TIS approach, has identified promising novel targets for drugs that can restore or enhance susceptibility to existing antibiotics. For example, analysis of insertion mutants spanning all non-essential *S. aureus* genes for fitness defects resulting from exposure to antibiotics, identified pathways/genes including *graRS* and *vraFG* (*graRS*/*vraFG*), *fmtA*, *mprF*, *SAOUHSC_01025*, and *SAOUHSC_01050* which if inhibited, can greatly improve the efficacy of existing antibiotics including daptomycin, vancomycin, gentamicin, ciprofloxacin, oxacillin, and linezolid and extend their utility for treating *S. aureus* infections (Rajagopal et al., 2016).

In the coming years, high-throughput next-generation strategies like TIS will undoubtedly rise to prominence in the NTM drug resistance field and radically advance our understanding of resistance mechanisms in *M. abscessus* and other emerging NTM pathogens. Such breakthrough technologies will greatly impact the kinds of questions which can be addressed in this field, for example, which resistance elements unique to *M. abscessus* render it unusually more drug resistant than other NTM species? or does *M. abscessus* already carry genes which confer resistance to novel NTM drugs in development?

## Author Contributions

All authors listed have made a substantial, direct and intellectual contribution to the work, and approved it for publication.

## Conflict of Interest Statement

The authors declare that the research was conducted in the absence of any commercial or financial relationships that could be construed as a potential conflict of interest. The reviewer UG and handling Editor declared their shared affiliation at time of review.
